# Action Being Character: A Promising Perspective on the Solution Concept of Game Theory

**DOI:** 10.1371/journal.pone.0019014

**Published:** 2011-05-09

**Authors:** Kuiying Deng, Tianguang Chu

**Affiliations:** 1 State Key Laboratory for Turbulence and Complex Systems, College of Engineering, Peking University, Beijing, China; 2 Key Laboratory of Machine Perception, Ministry of Education, Peking University, Beijing, China; Hungarian Academy of Sciences, Hungary

## Abstract

The inconsistency of predictions from solution concepts of conventional game theory with experimental observations is an enduring question. These solution concepts are based on the canonical rationality assumption that people are exclusively self-regarding utility maximizers. In this article, we think this assumption is problematic and, instead, assume that rational economic agents act as if they were maximizing their implicit utilities, which turns out to be a natural extension of the canonical rationality assumption. Implicit utility is defined by a player's character to reflect his personal weighting between cooperative, individualistic, and competitive social value orientations. The player who actually faces an implicit game chooses his strategy based on the common belief about the character distribution for a general player and the self-estimation of his own character, and he is not concerned about which strategies other players will choose and will never feel regret about his decision. It is shown by solving five paradigmatic games, the Dictator game, the Ultimatum game, the Prisoner's Dilemma game, the Public Goods game, and the Battle of the Sexes game, that the framework of implicit game and its corresponding solution concept, implicit equilibrium, based on this alternative assumption have potential for better explaining people's actual behaviors in social decision making situations.

## Introduction

Solution concepts of conventional game theory are mainly based on the canonical rationality assumption that players are rational economic agents who act as if they were maximizing their self-regarding utilities. Nevertheless, increasing experimental data support that decision makers in human society often do not act in accordance with predictions from these kinds of solutions [1]–[4]. This suggests that new insights and mechanisms are required to reconcile this undeniable inconsistency between theory and experiments. Indeed, similar cases often arise as well in many other areas of study such as physics. Galileo, founder of modern mechanics, proposed that all free falling bodies would fall through a vacuum with the same uniform acceleration, and also realized that, to explain why, as legend has it, the larger one of the two iron balls he dropped from the Tower of Pisa hit the ground slightly ahead of the smaller one, the resistance from air through which the balls were falling must be taken into account [5]. Similarly, the canonical rationality assumption provides a unified launching pad for a large body of theories in economics and related fields that work well to some extent and thus should not be given up lightly, but this does not imply that this assumption can be readily applied to explain people's actual behaviors without considering the effect of real game situations they are involved in, just as that of the air resistance on free falling balls.

So far, many efforts have been devoted to the modification or extension of the canonical rationality assumption in game theory with the aid of experimental observations. For instance, bounded rationality and other-regarding preference of players are considered in behavioral game theory [4], [6]. It is safe to say that people are not exclusively self-regarding [6], [7]. Costly punishment across human societies even in one-shot game situations is a fine example [8], [9]. People also show preferences for maximizing collective benefit or relative advantage over others' benefits in experiments [10], [11]. However, almost all experiments are designed to be simple and clear to make sure that all subjects have understood the rules completely [3], so there is little sense in trying to explain these experimental data by bounded rationality. Likewise, evolutionary game theory cannot explain why there are subjects in experiments who prefer to choose the strategy that is not evolutionarily stable when they clearly know this information, such as cooperating in one-shot Prisoner's Dilemma games [12]. The solution concept based on psychological game theory invokes psychological analysis about the motivations of other players [2]. In this article we will propose a promising solution concept which can not only avoid these vulnerabilities, but also provide better explanations of people's diverse behaviors unpredicted by conventional game theory based on the canonical rationality assumption.

Although the experimental evidence that rational self-regarding utility maximizers exist in chimpanzee's societies might offer some support for the canonical rationality assumption [13], many experimental results show that people are heterogeneous and behave differently when choosing rational strategies. The behaviors of decision makers in human society could be influenced by diverse factors such as sex [14], age [15], emotions [16], past experience [17], educational background [18], social distance [19], cultural difference [3], [20], and even experimental contexts [1]. For example, economists are more likely than others to free ride in public goods experiments [18], and there are the same players who converged to contribute nothing in a ten-period public goods experiment without punishment but contribute everything in a ten-period public goods experiment with punishment [21]. “Action is character”, wrote F. Scott Fitzgerald, author of The Great Gatsby, in one of his notebooks. What people do reflects what they are, and what people are implies what they do. We propose that rationality should be personal and related to the player's character. No matter how anomalous an action appears to be, it still should be thought as rational to those who take it after deliberation.

In this article we try to formally incorporate into the solution concept of game theory the player's character, a parameter encapsulating all diverse factors that may influence his decision making. The characters of players reflect their personal weighting between cooperative, individualistic, and competitive social value orientations. We assume that all players have a common belief about the character distribution for a general player, and rational players act as if they were maximizing their character-related utilities (that is, implicit utility we shall define later on), which turns out to be a natural extension of the canonical rationality assumption. In this procedure, a game is transformed into a Bayesian game, the solution of which, Bayesian Nash equilibrium, is referred to as implicit equilibrium of the original game. An implicit equilibrium provides players with a concrete decision rule which varies with their characters, and no player would feel regret about his decision even though the outcome turns out not to be a Nash equilibrium. The idea behind our framework turns out to be similar to the ERC model, a theory of equity, reciprocity, and competition [22]. But the ERC model uses equity as one of the primitive hypotheses and fails to incorporate cooperative factors, although it does consider players' heterogeneity and the tension between individualistic and competitive orientations [22]. In our framework, equity emerges in the Ultimatum game as a natural consequence of rational players acting as if they were maximizing their character-related utilities.

Solution concepts based on the canonical rational assumption also assume that it is common knowledge that each player is rational, each player knows that all players are rational, each player knows that each player knows that all players are rational, and so on *ad infinitum*. It is probably safe to say that this is not realistic in most cases. The concept of bounded rationality in behavioral game theory is proposed to avoid this vulnerability [6]. Instead, in our new framework of character and implicit equilibrium, we assume that the player facing a game situation chooses his strategy based on the common belief about the character distribution for a general player and the self-estimation of his own character, irrespective of what the characters of other players involved are, which strategies he think they will choose, and even whether they are rational or not. Rationality in our framework is only referred to as players maximizing their character-related utilities, and not as players with perfect memory and extraordinary computational capacity like supermen, and thus there is no need to invoke such concepts as bounded rationality. This contrast is one of the most important features of the concept of implicit equilibrium distinct from almost all other solution concepts proposed until now in game theory.

The concepts of character and implicit equilibrium we propose here based on this new rationality assumption can better explain people's actual behaviors labeled as irrational by conventional game theory in the experiments of some paradigmatic games. For example, why are at least one third of the dictators willing to offer the recipients more than zero in the Dictator game [23]? Why is it a modal pattern that two players end up with around fifty-fifty allocation of the pie in the Ultimatum game [3], [16]? Why are there subjects who cooperate in the Prisoner's Dilemma game [1] and contribute their endowments in the Public Goods game [3]? And why do people feel well satisfied with their choices even though they do not choose their own preferred programs simultaneously in the Battle of the Sexes game? All these puzzling but attractive questions are going to be given more reasonable explanations in our new framework based on the character-related rationality assumption that is presented subsequently.

## Analysis

We begin by defining an 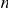-player noncooperative game in strategic form and, in passing, introducing the notations that will be used hereafter [24]. Let 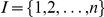 be the set of players, where 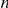 is the total number of players. For each player 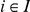, let 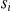 be a strategy of his and 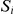 be the finite set of all his strategies. A vector 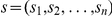 is called a strategy profile. The set of strategy profiles is thus the Cartesian product 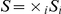. Let 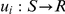 be the von Neumann-Morgenstern utility function associated to player 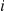 and 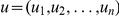 be the combined utility function of the game. Then a game in strategic form may be summarized as a triplet
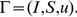
(1)


We decompose a player's utility into two parts: the outward-looking utility and the inward-looking utility. The former reflects the benefit of the group as a whole gained from playing the game and is the same for all players concerned, whereas the latter reflects the relative advantage of the focal player gained over the utilities of the other players involved in the game. A player's character is defined to reflect his personal weighting between the outward-looking utility and the inward-looking utility, or equivalently, his personal weighting between intergroup and intragroup competition.

Formally, for any strategy profile 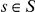, the *outward-looking utility* of player 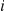 is defined as
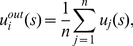
(2)and the *inward-looking utility*

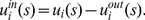
(3)Based on these two new concepts, we further define the *implicit utility* of player 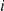 as
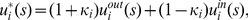
(4)where
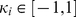
(5)represents player 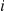
's *character* which reflects his personal weighting between the outward-looking utility and the inward-looking utility. When 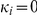, 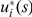 degenerates to 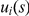. For the two-player case, 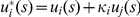 where 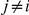, and for the zero-sum game where 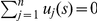, 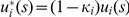. So implicit utility is defined by a kind of utility transformation in principle.

A player's character may take any value in the interval 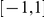 by definition. One with character 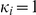 cares only about the outward-looking utility and acts by team-directed reasoning to achieve a group goal [25]. In this case the allocation of utilities among players is irrelevant to him. One with character 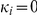 cares only about his self-regarding utility as in the canonical rationality assumption. This makes our new framework a natural extension of solution concepts in conventional game theory. One with character 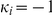 cares only about the inward-looking utility, that is, his advantage over other players. These three special cases reflect people's cooperative, individualistic, and competitive social value orientations, respectively, which form the theoretical basis for numerous studies [10].

Note that the inward-looking or outward-looking utility is distinct from the exclusively self-regarding or other-regarding utility. This is an issue about what factors should be considered as the primitive assumptions about people's instincts related to decision making. We think that the exclusively other-regarding preference as a primitive assumption might contradict the principle of individuals being the unit of natural selection. The definition of implicit utility in (4) is equivalent to
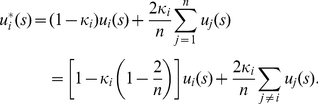
(6)This implies that any player 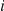, regardless of his character 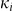, always gives a positive weighting to his exclusively self-regarding utility 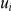, and thus he will never show an exclusively other-regarding preference by definition. Actually, the weighting player 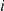 gives 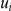 is always more than that he gives 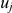, where 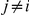, by 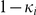.

The character of a player, which reflects his personality when facing a game situation, may vary with the contexts in which he needs to make a decision. For example, experimental data show that the cooperation rate usually decreases in repeated public goods games without punishment relative to in those games with punishment [21], which reveals that players might update their characters by learning to adapt to particular situations. However, considering the fact that players from some specific social context usually follow the same code of conduct, whether religious, ethical, or legal, we assume that,

*when facing one-shot game situations without communication, all players have a relatively stable common belief* 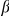 *about the character distribution for a general player over the interval* 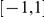.

In other words, for any focal player, 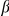 is the best of his knowledge about other players in a one-shot game situation. As far as the real world is concerned, we think that it might be inappropriate to always assume that people who face a one-shot game situation have no knowledge of the characters of other players involved.

By incorporating players' characters and their common belief about the character distribution for a general player, any game can be transformed into a Bayesian game. In particular, let 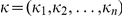 and 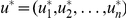. Then a Bayesian game
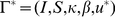
(7)can be defined. We call 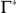 the *implicit game* corresponding to 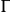, and its Bayesian Nash equilibrium,
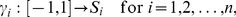
(8)the *implicit equilibrium* of 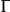, which provides the player with a concrete decision rule based on the self-estimation of his own character, irrespective of other players' characters. No matter whether the outcome turns out to be a Nash equilibrium of the game 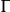 or not, no player would feel regret about his decision and thus no player would want to change it.

In contrast to the canonical rationality assumption, the idea underlying this transformation is referred to as the *character-related rationality assumption*; that is,

*all players are rational economic agents who act as if they were maximizing their implicit utilities*.

For those players with character 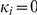, this is exactly the canonical rationality assumption, except that we do not need the assumption that rationality is common knowledge. We expect that the concepts of implicit game and implicit equilibrium based on this alternative assumption can better model how decisions are made in actual social contexts. Subsequently we apply these new concepts to some paradigmatic game situations to show their capability and effectiveness.

### The Dictator Game

The Dictator game is about sharing a pie between two anonymous players, in which the dictator who has complete control over the process of allocation chooses to offer a portion of the pie, 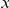, to the recipient who has no choice but to accept it, and thus leaves 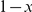 to himself (Table 1). This one-shot game is designed to measure the altruism behavior of the dictator, which is not directly associated with other plausible factors such as kinship, reciprocity, reputation, or the immediate threat of punishment [8].

**Table 1:** The Dictator Game.

Dictator	RecipientAccept
Offer 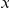	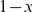 , 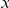

According to the canonical rationality assumption, the rational dictator who acts as if maximizing his self-regarding utility would take advantage of his dominant position and thus choose to offer the recipient nothing. However, a consistent observation in experiments is that at least one third of the dictators are willing to offer the recipients more than zero across a wide variety of procedures and conditions, although it is true that most of the dictators offer nothing [23].

In contrast, the character-related rationality assumption predicts that not all the dictators who act as if maximizing their implicit utilities would offer nothing, as shown in the following proposition.

#### Proposition 1

*In the Dictator game, (i) a rational dictator with character* 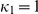 *would like to offer the recipient any portion of the pie, even as big as the whole pie, and (ii) a rational dictator with character* 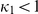 *would offer the recipient nothing*.

#### Proof

Suppose that the dictator, player 1, offers a portion of the pie, 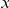, to the recipient, player 2, and leaves 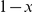 to himself. The implicit utility of player 1 is
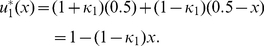
(9)


If 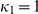 then 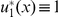. That is, the implicit utility of the dictator with character 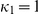 is constantly equal to 1 whatever he gets from the allocation of the pie. So a rational dictator with character 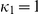 would like to offer any portion of the pie 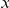 to the recipient, even as big as the whole pie.

If 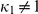 then 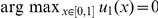 and 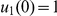. That is, a rational dictator with character 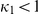 who try to maximize his implicit utility would offer the recipient nothing so as to get the maximum implicit utility 1.

As a result of the recipient's inability to influence the allocation procedure, the implicit equilibrium presented in this proposition does not even need the dictator and the recipient to have a common belief 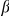 about the character distribution for a general player.

It is worth noting that we can get 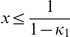 by solving 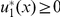 in the proof above, which means that the dictator with character 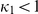 would still be better off if he offers the recipient 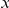 not more than 
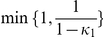
 than not participating in the game, even though he does not manage to maximize his implicit utility due to some unknown reasons. In particular, those dictators with non-negative characters who fail to maximize their implicit utilities could be better off even though they offer the whole pie. This point may be helpful to the understanding and explanation of why there are the dictators who are willing to offer more than zero in experiments, except that those dictators with character 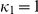 who care only about the collective benefit would anyway.

### The Ultimatum Game

The Ultimatum game is similar to the Dictator game. One only difference is that, in the Ultimatum game, after the proposer offers a portion of the pie, 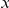, to the responder and leaves 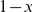 to himself, the responder could choose either to accept it with the allocation of the pie settled down or to reject it with two players getting nothing (Table 2) [8]. The responder in this one-shot anonymous game is endowed with an opportunity to punish the proposer's greed, but, if he did, what he does would cost him all he could own.

**Table 2:** The Ultimatum Game.

Proposer	Responder	
	Accept	Reject
Offer 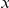	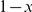 , 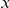	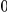 , 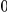

According to the canonical rationality assumption, this game has a subgame perfect Nash equilibrium where the proposer offers the smallest nonzero amount and the responder accepts it, because a rational proposer knows that the responder who maximizing his self-regarding utility will always accept any positive offer [8]. However, experimental evidence shows that (i) the offer 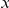 distributes over the whole interval 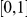 [3], (ii) offers above 0.5 are rare in student subjects [26], (iii) a modal pattern is that two players end up with around fifty-fifty allocation of the pie [3], [16], and (iv) there are the responders who accept the smallest nonzero offer and the proposers who offer the whole pie [3].

In contrast, the character-related rationality assumption that players act as if they were maximizing their implicit utilities provides a more reasonable explanation for all these experimental observations.

#### Proposition 2

*In the Ultimatum game, (i) a rational proposer with character* 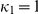 *would like to offer any portion of the pie not less than* 
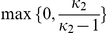
 *, even as big as the whole pie, where* 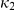 *is the character of the responder, and the responder would accept it, and (ii) a rational proposer with character* 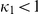 *would offer* 
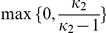
 *which is never more than half the pie, and the responder would accept it*.

#### Proof

Suppose that the proposer, player 1, offers a portion of the pie, 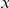, to the responder, player 2, and leaves 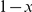 to himself. The implicit utility of player 1 is
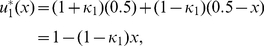
(10)and the implicit utility of player 2 is
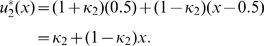
(11)If an offer is accepted by both players, 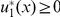 and 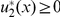 are needed to be satisfied simultaneously. Solving these two inequalities, we get
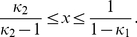
(12)So an offer 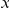 that exits between 
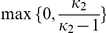
 and 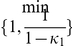 would be accepted by both players.

Because the implicit utility 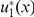 of a proposer with character 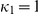 is constantly equal to 1 whatever he gets from the allocation of the pie, his rational strategy is to offer any 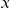 that will be accepted by the responder, that is, not less than 
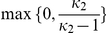
, even as big as the whole pie.

The implicit utility 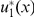 of a proposer with character 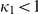 increases with 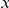 decreasing, so he would like to offer the lower bound of the offer that the responder will accept so as to maximize his implicit utility, that is, 
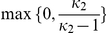
. Since 
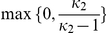
 increases with 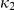 decreasing and reaches the maximum 0.5 when 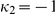, a rational proposer with character 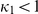 would never offer the responder more than half the pie.

From the proof above we can also infer that the responder with non-negative character would accept any offer 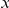 by the proposer, since the lower bound 
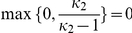
 when 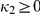. Furthermore, since the upper bound 
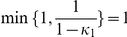
 when 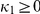, the proposer with non-negative character might offer the responder any 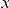, even as big as the whole pie. In these two cases players would be better off than not participating in the game, even if they do not manage to maximize his implicit utility due to some unknown reasons. These two points might be helpful to explain why, in experiments, the offer 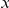 distributes over the whole interval 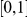 and there are the responders who accept the smallest nonzero offer and the proposers who offer the whole pie.

As in the Dictator game, the implicit equilibrium presented in this proposition does not also require the proposer and the responder to have a common belief 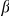 about the character distribution for a general player, because of the asymmetrical positions of the two players in the allocation procedure. However, close inspection will reveal that this implicit equilibrium is inconsistent with our aforementioned presentation that, when a player who acts as if maximizing his implicit utility chooses a strategy, the characters of other players are irrelevant. The strategy we give here requires the proposer to estimate the character of the responder.

Let us dig deeper into the procedure of the proof of this proposition. The upper bound of the offer that both players will accept, 
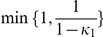
, decreases with the character of the proposer 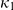 decreasing and reaches the minimum 0.5 when 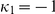, while the lower bound, 
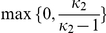
, increases with the character of the responder 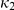 decreasing and reaches the maximum 0.5 when 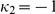. If we think of student proposers as players who care only about their inward-looking utility and are good at estimating responders' characters, then this analysis can explain why offers above 0.5 are rare in student subjects in experiments. This analysis also gives us a far-reaching conclusion as follows.

#### Proposition 3

*In the Ultimatum game, half the pie is the unique offer that would be always accepted by any two players*.

Now the characters of both the proposer and the responder fade out completely. Actually, in the case where 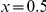, solving 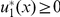 and 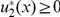 gives us 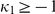 and 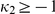, which can be satisfied by any two players by definition. This proposition finally provides us with a perfect explanation of why around fifty-fifty allocation of the pie is observed as a modal pattern in experiments.

Fairness, or inequality aversion, is recognized as one of key elements in human strategic interactions [16]. Here we show that the proposer giving half the pie and the responder accepting it is an implicit equilibrium, irrespective of the characters of the proposer and the responder. Hence, fairness is achieved as a natural consequence of two players acting as if they were maximizing their implicit utilities, whereas in almost all other existing theoretical models it is incorporated explicitly as one of primitive assumptions about people's instincts related to decision making [10], [22], [26], [27].

### The Prisoner's Dilemma Game

The best known model in game theory might be the Prisoner's Dilemma game, which is also played between two anonymous players (Table 3). Each player in this game can either cooperate or defect. One cooperating would induce cost 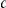 to himself and bring benefit 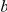 to the other. One defecting costs nothing and benefits neither. Note that 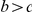 is usually assumed so as to make sure that two cooperating players can benefit more than two defecting players. This simple model and its finitely and infinitely repeated versions are extensively used to explore the mechanism of the evolution of cooperation through natural selection [28].

**Table 3:** The Prisoner's Dilemma Game.

Focal player	Another player	
	Cooperate (C)	Defect (D)
Cooperate (C)	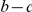	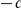
Defect (D)	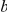	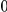

According to the canonical rationality assumption, one in this scenario would always defect since defecting is a dominant strategy. That is, one can always be better off by defecting than by cooperating, whether the other player defects or cooperates. By backward induction, we know, even in the finitely repeated version, defecting in all steps is the best choice. However, each could achieve a higher utility 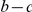 by both cooperating than 0 by both defecting. This is why the Prisoner's Dilemma game is often used as a synonym for “social dilemma”.

The predictions given by the canonical rationality assumption contradicts experimental data. There are subjects who cooperate even in the last round of finitely repeated situations, and the experimental studies from 1958 to 1992 show that the cooperation rate of players ranges from 5% to 96.9%, with a mean of 47.4% [1]. In contrast, by the character-related rationality assumption, one with character 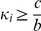 would cooperate in the Prisoner's Dilemma game so as to maximize his implicit utility.

#### Proposition 4

*In the Prisoner's Dilemma game, one would cooperate if his character* 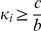 *, and otherwise defect, given that the two players have any common belief about the character distribution for a general player*.

#### Proof

For player 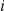 with character 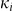 in the Prisoner's Dilemma game, when he cooperates, his implicit utility is 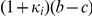 if the other player cooperates and otherwise 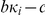 if the other player defects. When he defects, his implicit utility is 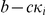 if the other player cooperates and otherwise 0 if the other player defects.

We assume that the two players have a common belief 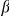 about the character distribution for a general player and the character of a player who cooperates is not less than a critical value 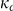. Then, the expected implicit utility of player 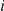 cooperating is
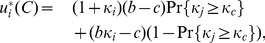
(13)where 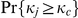 is the probability of the other player 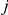 cooperating, and the expected implicit utility of player 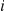 defecting is
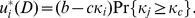
(14)Solving 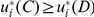, we get 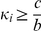. So 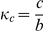. In other words, it is an implicit equilibrium that a player cooperates when his character 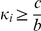, and otherwise defects.

This implicit equilibrium is symmetric in the sense that it works for either player. No player would feel regret even if he chooses to cooperate and the other player chooses to defect, although this outcome is not a Nash equilibrium. From this implicit equilibrium we can also infer that the players would tend to defect when 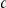 is near 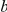, because the portion of people with character 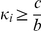 becomes far less whatever the character distribution for a general player is.

Cooperation is considered as a third fundamental principle of evolution beside mutation and natural selection [29]. The repeated Prisoner's Dilemma game is used to describe cooperation between unrelated individuals. If the probability of another encounter between the same individuals exceeds the cost-to-benefit ratio 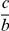, cooperation can evolve [29]. Now this proposition identifies 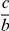 as an important parameter in the one-shot Prisoner's Dilemma game, either. If the player self-estimates that his character exceeds 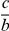 then he would cooperate. These two conclusions complement each other perfectly.

### The Public Goods Game

In the Public Goods game, each of 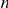 players is endowed with one unit of utility and secretly decides whether to contribute it into the public pot or to free ride. The umpire will multiply the sum of contributions in the pot by a number 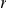 greater than 1 and smaller than 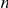, and then split the total utility equally among all players, irrespective of whether they contribute or not (Table 4) [30]. This game can be considered as an extension of the Prisoner's Dilemma game to the 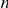-player situation [30]. In particular, when there are only two players, the Public Goods game is equivalent to the Prisoner's Dilemma game with 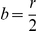 and 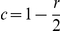.

**Table 4:** The Public Goods Game.

Focal player	Other 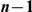 players 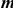 contribute (C)
Contribute (C)	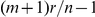
Free ride (D)	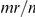

According to the canonical rationality assumption, a rational player in this scenario would free ride since free riding is a dominant strategy. One will incur a loss of 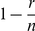 units of utility by contributing whatever others do. However, each of 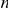 players could benefit most and get utility 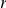 from all players contributing. This makes it a social dilemma essentially the same as the Prisoner's Dilemma game.

In the Public Goods game experiments where players are permitted to contribute any fraction of their endowments, the mean contributions usually end up with between 40% and 60% [3]. Although the definition of the Public Goods game in these experiments is not exactly the same as that given above, the data surely reflects that those subjects are not exclusively self-regarding utility maximizer. By the character-related rationality assumption that players act as if they were maximizing their implicit utilities, we may give a more reasonable solution to the Public Goods game defined here.

#### Proposition 5

*In the Public Goods game, one would contribute his endowment if his character* 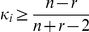 *, and otherwise he would free ride, given that all players have any common belief about the character distribution for a general player*.

#### Proof

We use 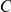 and 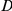 to denote two strategies of the player, contributing his endowment and free riding, respectively. For any given player 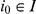, when there are 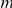 players using strategy 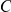, the outward-looking utility of player 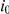 using strategy 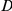 is
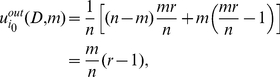
(15)and the inward-looking utility is
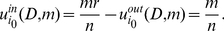
(16)Then we can work out the implicit utility of player 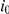 using strategy 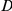 when there are 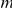 players using strategy 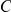, which is
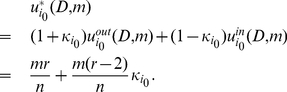
(17)We assume that all 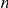 players have a common belief 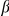 about the character distribution for a general player and the character of a player using strategy 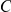 is not less than a critical value 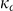. Hence the expected implicit utility of player 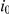 using strategy 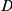 is
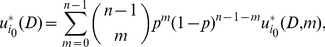
(18)where 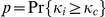 is the probability of any player 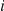 using strategy 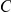.

For any given player 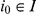, when there are 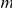 players using strategy 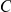, the outward-looking utility of player 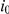 using strategy 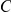 is
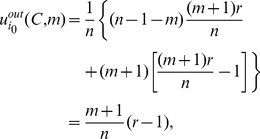
(19)and the inward-looking utility is
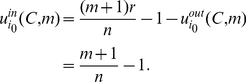
(20)Then we can work out the implicit utility of player 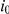 using strategy 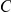 when there are 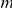 players using strategy 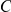, which is
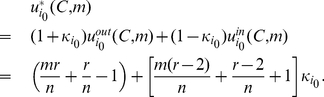
(21)Hence the expected implicit utility of player 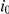 using strategy 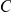 is
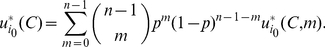
(22)


For solving 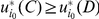, we calculate
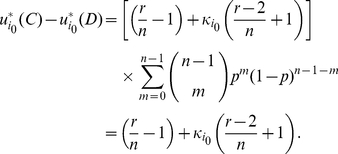
(23)Then we get 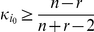 by solving 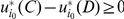. So 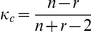. That is, it is an implicit equilibrium that a player would contribute his endowment if his character 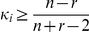, and otherwise he would free ride.

The implicit equilibrium in this proposition is also symmetric and works for all players. For the two-player case, the critical value 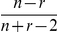 is consistent with 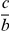 in the Prisoner's Dilemma game with 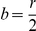 and 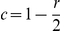. Note that the limit of 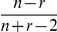 is 1 as 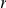 approaches 1 or 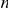 is very large relative to 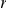, so the probability of players contributing his endowment is extremely low. In other words, the player tends to free ride when the multiplication factor 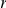 is small or the number of the players involved, 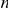, is large.

Note that the implicit equilibria of the Dictator game and the Ultimatum game are not related to the players' common belief 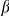 about the character distribution for a general player. The implicit equilibria of the Prisoner's Dilemma game and the Public Goods game do, but not to what the common belief 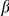 itself is. In general these are not the case, for instance, as shown below in the Battle of the Sexes game.

### The Battle of the Sexes Game

In the Battle of the Sexes game [2], one of two anonymous players prefers to watch soccer, whereas the other prefers to watch opera. Each player can get one unit of utility by watching his preferred program and additional 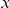 units by getting the other for company (Table 5). When 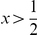, these two players as a whole will be better off by watching the same program, either soccer or opera, than by watching different ones. This game is a formal representation of coordination problems which are widespread in life, such as choosing which side of the road to drive and what gifts to exchange like the young couple in the story of The Gift of the Magi by O. Henry. Another well-known game that essentially has the same coordination nature as the Battle of the Sexes game is the Stag Hunt game [2].

**Table 5:** The Battle of the Sexes Game.

Focal player	Another player	
	Opera (Own)	Soccer (Other)
Soccer (Own)	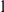	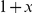
Opera (Other)	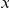	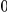

According to the canonical rationality assumption, this game has two pure strategy Nash equilibria: both watching soccer or both watching opera. In addition, this game has a mixed strategy Nash equilibrium; that is, each player watches his preferred program with probability 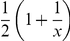 and the other player's preferred program with probability 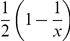. When this game is played once without communication, multiple Nash equilibria can not give players much instruction, and disequilibrium may result. In contrast, the character-related rationality assumption that players act as if they were maximizing their implicit utilities can provide players with a more concrete strategy of play, given that they have a common belief 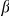 about the character distribution for a general player.

#### Proposition 6

*In the Battle of the Sexes game, one would watch the other player's preferred program if his character* 
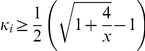
 *, and otherwise his own preferred program, given that both players believe that the character of a general player is uniformly distributed over the interval* 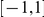.

#### Proof

For two players, 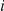 and 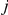, in the Battle of the Sexes game, when player 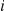 watches his preferred program, his implicit utility is 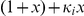 if player 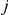 accompanies him and otherwise 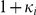 if player 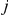 watches his own preferred program. When player 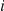 watches player 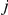
's preferred program, his implicit utility is 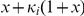 if player 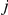 accompanies him and otherwise 0 if player 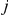 watches player 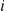
's preferred program.

We assume that the two players have a common belief 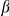 about the character distribution for a general player and player 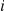 would watch player 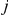
's preferred program when 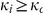. The expected implicit utility of player 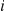 watching the other player 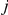
's preferred program is
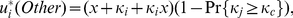
(24)where 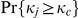 is the probability of player 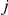 watching player 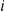
's preferred program. The expected implicit utility of player 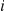 watching his own preferred program is
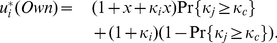
(25)Solving 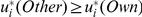 gives us
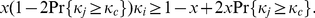
(26)


Further we assume that both players believe that the character of a general player is uniformly distributed over the interval 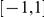, which leads to 
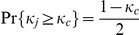
. Substituting it into the above inequality gives us
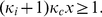
(27)Considering that 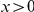 and 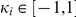, one necessary condition for this inequality to be justifiable is 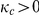. So we can get
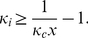
(28)Setting 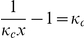 and solving it give us 
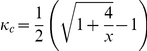
. Thus it is an implicit equilibrium that one would watch the other player's preferred program if his character 
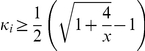
, and otherwise he would watch his own preferred program.

The procedure of proof shows that the implicit equilibrium in the Battle of the Sexes game is closely related to the players' common belief 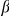 about the character distribution for a general player. As an alternative, if we assume that both players believe that the character of a player is uniformly distributed over the interval 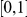, then 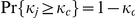. Then the same procedure as in the proof above leads to 
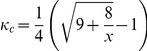
. Consequently, we arrive at the following conclusion.

#### Proposition 7

*In the Battle of the Sexes game, one would watch the other player's preferred program if his character* 
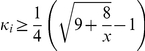
 *, and otherwise his own preferred program, given that both players believe that the character of a general player is uniformly distributed over the interval* 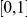.

Under both assumptions about the players' common belief 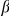 about the character distribution for a general player, the implicit equilibria are symmetric and works for either player. Further, we can expect that one will watch the other player's preferred program with probability 
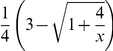
 in the first case, and with probability 
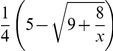
 in the second case. Yet we have to emphasize that no player feels regret in implicit equilibrium even if they watch different programs, that is, they simultaneously choose to watch their own or the other player's preferred programs. This is totally distinct from Nash equilibrium where both players watching different programs is considered as disequilibrium in which they feel regret and want to change their strategies.

When additional utility 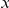 from the company of the other player is extremely large (or extremely small), the implicit equilibria in both cases predict that the probability of one watching the other player's preferred program would approach 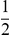 (or 0). These are consistent with the predictions of the mixed strategy Nash equilibrium which says this probability is 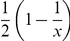. Nevertheless, when 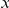 is between 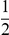 and 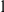, the three Nash equilibria degenerate to one where each player would watch his own preferred program with certain, although these two players as a whole can still be better off by both watching soccer or both watching opera. In contrast, we can expect that one will watch the other player's preferred program with positive probability by the predictions of the implicit equilibria under both assumptions above. This point adds to the evidence that the implicit equilibrium might be more reasonable than the three Nash equilibria in the Battle of the Sexes game.

## Results and Discussion

The inconsistency of predictions from solution concepts of conventional game theory with experimental observations is an enduring question. By clearly taking the player's heterogeneity into consideration, we have shown in five paradigmatic games, the Dictator game, the Ultimatum game, the Prisoner's Dilemma game, the Public Goods game, and the Battle of the Sexes game, that the concepts of implicit game and implicit equilibrium have potential for better explaining people's actual behaviors in social decision making situations. This theoretical framework is based on the so-called character-related rationality assumption that rational economic agents act as if they were trying to maximize their implicit utilities. The cooperative, individualistic, and competitive social value orientations are incorporated as three special cases where the player's character equals 1, 0, and 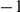, respectively [10]. The player chooses his strategy based on the common belief about the character distribution for a general player and the self-estimation of his own character, and he is not concerned about which strategies other players will choose and will never feel regret about his decision.

The concept of character reflects players' heterogeneity, although it does not explicitly consider complicated psychological motivations as in psychological game theory [2]. The ERC model does consider players' heterogeneity and the tension between individualistic and competitive orientations, but fails to incorporate cooperative factors [22]. The concept of implicit utility is basically a kind of utility transformation, but it is based on the player's self-estimation of his own character, rather than on some inflexible parameters that are supposed to be imposed on all players as in other models [10]. We suppose that the character-related rationality assumption as a natural extension of the canonical rationality assumption is the consequence of evolution, and surely we do not explicitly consider its evolutionary process as in evolutionary game theory [12] or bounded rationality as in behavioral game theory [6].

However, as aforementioned, a player's character could be influenced by so many factors that it seems to be a big question how to measure it. Even if this is the case, incorporating the player's character in analyzing games would no doubt be useful to explain people's diverse behaviors in decision making as we shown here. At this point it may be suitable to mention another fact from physics. The temperature of an object is also influenced by many complicated factors, yet now we can conveniently measure it using a thermometer. Likewise, constructing a proper procedure, or even a convenient device, to measure the character of a player facing a game situation is a promising and challenging project. Another interesting issue is how the character of a player (consequently his strategy) evolves in repeated game situations with new information about the strategies of other players in previous plays. The progress in either direction would further extend our understanding about the reason people cooperate in social dilemmas and the way decisions are actually made.
